# 

**Published:** 1861-06

**Authors:** 


### CONTENTS.---JUNE, 1861.

### ORIGINAL CONTRIBUTIONS.

PAGE

ART. I. Lectures on Military Surgery. By E. ANDREWS, A. M., M. D., Professor of Surgery, etc. Lecture II., ........ 257

ART. II. Comments on "Notes relating to the Extirpation of the Parotid Gland," by Prof. Brainard, M. D. Remarks on the Surgical Treatment of Spina Bifida—Summary of Prof. Nelaton's Clinical Lecture on the Injection of Solution of Iodine in cases of Hydrorachitis, after the manner employed by Prof. Brainard. By TITUS DEVILLE, M. D., ........ 264

ART. III. The Miracle of Anæsthesia. An Inaugural Thesis. By FRANK W. REILLY, M. D., ........ 269

### THE CLINIQUE.

MERCY HOSPITAL—Surgical Wards—Service, Prof. E. ANDREWS, M. D. Excision of the Head of the Femur, ........ 292

Fistula from the Hip Joint into the Rectum, ........ 293

Splints for Hip Disease, ........ 294

MARINE HOSPITAL.—Service, Prof. R. N. ISHAM, M. D.; reported by STACY HEMENWAY, Student of Medicine. Skin Diseases, ........ 295

Aneurism of Aorta with Cardiac Hypertrophy, ........ 297

Fracture of the Acromion Process, ........ 300

### SELECTIONS.

Directions to Army Surgeons on the Field of Battle. By Surgeon-General G. J. GUTHRIE, 302

Prevention of Vomiting after Inhalation of Chloroform.—FISCHER, ........ 291

Tannin an Antidote of Strychnia.—KURSAK, ........ 301

### BOOK NOTICES.

A Practical Treatise on Phthisis Pulmonalis. By L. M. LAWSON, M. D., Prof. Clin. Med. Univ. of La., ........ 307

Forty-Fourth Annual Report of the Friends' Asylum, near Philadelphia.....Annual Report of the Longview, Ohio, Asylum, ........ 312

Report of the Board of Health of Philadelphia, 1860 ........ 313

Hand-Book for the Military Surgeon. By CHARLES S. TRIPLER, A. M., M. D., U. S. A.. and GEORGE C. BLACKMAN, M. D., F. R. M. S., Professor of Surgery in the Medical College of Ohio, etc., ........ 314

A Report of Epidemics and Endemics—Cholera. By O. C. GIBBS, M. D., Frewsburg, New York, ........ 315

A Manual of Military Surgery. By S. D. GROSS, M. D., Professor of Surgery in the Jefferson Medical College, Philadelphia, ........ 316

Practical Treatise on Military Surgery. By FRANK HASTINGS HAMILTON, M. D., ........ 316

### EDITORIAL.

Items.—A Miscalculation....To the Medical Staff, U. S. A....Illinois Physicians in Michigan....Summer Schools,,..Contagiousness of Diphtheria, ........ 317

Obituary—Dr. Lawson....Dr. Reese ........ 318

Scurvy ........ 319

Special Notices ........ 319

